# Editorial: Extreme Environments in Movement Science and Sport Psychology

**DOI:** 10.3389/fpsyg.2018.02391

**Published:** 2018-12-04

**Authors:** Costantino Balestra, Jacek Kot, Shai Efrati, François Guerrero, Jean-Eric Blatteau, Stéphane Besnard

**Affiliations:** ^1^Environmental, Occupational and Ageing “Integrative Physiology” Laboratory, Haute Ecole Bruxelles-Brabant (HE2B), Brussels, Belgium; ^2^Faculté des Sciences de la Motricité, Université Libre de Bruxelles, Brussels, Belgium; ^3^DAN Europe Research Division, DAN Europe Research (Roseto-Brussels), Brussels, Belgium; ^4^National Centre for Hyperbaric Medicine, Institute of Maritime and Tropical Medicine, Medical University of Gdansk, Gdynia, Poland; ^5^Sagol Center for Hyperbaric Medicine and Research, Assaf-Harofeh Medical Center, Tzrifin, Israel; ^6^Sackler School of Medicine and Sagol School of Neuroscience, Tel-Aviv University, Tel Aviv, Israel; ^7^EA4324 ORPHY, Institut Brestois Santé Agro Matiére, Université de Bretagne Occidentale, Brest, France; ^8^Hôpital d'Instruction des Armées - Service de Médecine Hyperbare et Expertise Plongée (Military Teaching Hospital - Hyperbaric Medicine and Diving Expertise Department), Toulon, France; ^9^UNICAEN, INSERM U1075, COMETE, Normandie Université, Caen, France

**Keywords:** unexpected results, challenging environments, neurosciences, integrative approach, adaptive

If we want simply to depict what extreme environments are, we can consider them as primarily depending on two parameters: temperature and pressure. Gravitational and radiation changes can both also be added.

As a matter of fact, both dimensions are also well-linked together. Depending on those two parameters, hydration, gas partial pressures, effort, work of breathing, metabolism, gene expression and many other essential “ingredients” of human life and performance can vary widely.

Human studies in extreme environments (altitude hypoxia, microgravity, hyperbaric, and terrestrial extreme climatic conditions) over the last decades have expanded knowledge in physiology, highlighting new routes of regulation, breaking previous old concepts, and offering new models of some physiopathological troubles in patients (Trivella et al., 2017; Burtscher et al., 2018).

Some years ago, on the physiological side, the two parameters that characterize extreme environments were identified to elicit the production of two particular elements: Hypoxia inducible factors and heat shock proteins. Surprisingly, these two elements can be triggered by either hypobaric/hypoxic or hyperbaric/hyperoxic environments. The reason is that, in biologym what is mostly being sensed is the fluctuation rather than absolute values.

The two are ubiquitous and essential to cellular life. The first is a factor that triggers around 200 genes responsible for vascular, cellular, and metabolic homeostasis as well as apoptosis. In fact, its beneficial actions on the fight against cancer cells have recently been advocated (De Bels et al., 2011; Khalife et al., 2018). The second is a family of proteins acting as chaperones for other proteins and resetting impaired proteic structures (Kopecek et al., 2001; Gjovaag and Dahl, 2006; Hageman et al., 2011).

Some psychological aspects have been explored independently or sometimes combined with physiology. However, little is known about cognition and neuronal plasticity in extreme environments, although adaptation to extremes is an integrative matter that the body and brain have to solve conjointly. How do peripheral body signals, homeothermic regulation, energy expenditure, and psychological and cognitive functions interact with each other? New insights on how extreme external factors may change emotional and cognitive functions of self-perception and the perception of the surrounding environment, and what impact this has on decision-making processes, are matters of interest.

It has long been established that a general law applies to humans in extreme environments: the Yerkes-Dodson Law (Calabrese, 2008). The relationship between arousal and performance is known and discussed (Balestra et al., 2018), but we seldomly know up to what point the positive effect on performance/coping exists in extreme environments (Mair et al., 2011; Rietschel et al., 2011).

Not so long ago it has been shown that environments are also able to interact with the genome. In fact, epigenetics seems to be a major point in extreme environments, especially when partial oxygen pressure changes are involved (Lautridou et al., 2017b; Kiboub et al., 2018b), but remains poorly investigated.

The proposed research topic has addressed most of the psychological and physiological reflexions needed in extreme environments, opening for future research and progress.

New challenges are also important in changing gravity environments. Although physiological and psychological parameters have been widely investigated, cognitive functions during long term missions in space remain to be evaluated. For example, spatial cognition, including the self-perception, orientation and navigation required during 3D robotic arm control, rendez-vous docking and extra-vehicular activities are all affected by the loss of gravity-related sensors. Koppelmans et al. (2013) consequently, the next challenging step is understanding how decision making, spatial cognition, emotional aspects, as well as cortical sensory integration supporting self-bodily perception and orientation are influenced by and during extreme short or prolonged missions. The ways humans have adapted ancestrally and how we will adapt to strong and fast environmental and climatic changes on Earth require an integrative approach at the frontiers between cognition, psychology, and physiology. Reviews, reports, and the most recent data will support the preparation for human solar system exploration, firstly to Mars. Understanding how humans cope with extreme environmental or physiological/psychological challenges has helped us to leave our comfortable paradigms built on stable “steady states” (Balestra, 2012). Today's measurement systems allow us to analyze our reactions to intermittent stressors and follow the oscillations of our coping mechanisms. This new approach has led us to unexpected understandings (Lautridou et al., 2017a) since most of the results expressed in this research topic are unexpected or even counterintuitive.

This methodology has also directly improved our translational or multidisciplinary (integrative) approach as well as the idea that studying humans in good health at extremes could help us to understand both patients (Khalife et al., 2018; Kiboub et al., 2018a) with impaired physiological capacities coping with our environment (which) becomes extreme to them), or better understanding physiology/psychology of the elderly, or to better prepare people working in constraining environments.

## Author Contributions

All authors listed have made a substantial, direct and intellectual contribution to the work, and approved it for publication.

### Conflict of Interest Statement

The authors declare that the research was conducted in the absence of any commercial or financial relationships that could be construed as a potential conflict of interest.
